# Assessing Short‐term Memory with Different Memory Modalities in Mild Cognitive Impairment

**DOI:** 10.1002/alz.084723

**Published:** 2025-01-03

**Authors:** Elaina Smith, Christopher Cortez, April R Wiechmann, Sandra Davis, Hannah Dyson, Krystyn Kucharski, Sarah M Ross, Geoffrey P Kline, Robert T Mallet, Xiangrong Shi

**Affiliations:** ^1^ University of North Texas Health Science Center, Fort Worth, TX USA

## Abstract

**Background:**

Mild cognitive function (MCI) is associated with a declined short‐term memory (STM). This study compared STM between adults with MCI and normal cognition assessed by verbal memory vs visuospatial memory.

**Methods:**

Sixteen subjects with MCI and 11 subjects with normal cognition gave their written consent to participate in the study which was approved by the North Texas Regional IRB. Subjects having a self‐ or family member‐reported memory complaint, whose clinical dementia rating was ≤0.5, and/or whose testing scores in two or more cognitive domains were below the age‐/education‐adjusted group averages, were determined to have MCI. Digit‐Span‐Test (DST) and California‐Verbal‐Learning‐Test (CVLT‐II) were assessed for digit‐verbal memory and word‐verbal memory, respectively. Brief‐Visuospatial‐Memory‐Test–Revised (BVMT‐R) was performed for visuospatial memory. Values from the MCI and normal groups were compared using t‐tests. Two‐factor ANOVA was applied to test the significance of the group factor (*i.e*., MCI vs normal) and the trial factor (*i.e*., trials 1‐4 in CVLT‐II and trials 1‐3 in BVMT‐R).

**Results:**

Neither group age nor education attainment was different in MCI vs normal (71.3±1.6 vs 67.9±1.7 years old). Although MMSE scores were not different between the groups, Trail‐Making‐Test performance was significantly poorer in the MCI. DST‐Sequencing scores were lower (P = 0.011) in the MCI (4.8±0.4) vs normal (6.4±0.3) subjects. However, neither DST‐Forward nor DST‐Backward scores differed between the groups. CVLT‐II immediate free‐recall and BVMT‐R recall scores were consistently superior in the normal vs MCI subjects (group factor P<0.001) and improved significantly with trial repetitions (trial factor P<0.001) in both groups. The rates of performance improvement with repeated CVLT‐II and BVMT‐R trials were similar in the groups, indicating similar learning effects. Both 30‐s short‐delayed and 10‐min long‐delayed free‐recall scores in CVLT‐II and 30‐min delayed recall scores in BVMT‐R were significantly lower in the MCI vs normal subjects (CVLT‐II short‐delayed: 6.9±0.3 vs 8.4±0.2 [P<0.001]; long‐delayed: 5.9±0.4 vs 8.2±0.3 [P<0.001], and BVMT‐R delayed recall: 5.1±0.9 vs 7.9±0.5, [P = 0.021]).

**Conclusions:**

Both the verbal memory and visuospatial memory are significantly diminished, but learning ability may be preserved in MCI. CVLT‐II seems to be more specific and/or sensitive for detecting MCI‐related difference in STM.

